# Supplementary material for: Translation, Cross-Cultural Adaptation, and Psychometric Properties of the Polish Version of the Hip Disability and Osteoarthritis Outcome Score (HOOS)
Source: Medicina (Kaunas). 2019 Sep 20;55(10):614. doi: 10.3390/medicina55100614 (PMC6843159; doi:10.3390/medicina55100614)
Supplement: Supplementary file 1 [file medicina-55-00614-s001.pdf]

# HOOS - KWESTIONARIUSZ DLA CHORYCH Z DOLEGLIWOŚCIAMI STAWU BIODROWEGO

Dzisiejsza data ____/____/________ Data urodzenia ____/____/________

Imię i nazwisko ______________________________________________

**INSTRUKCJA:** Niniejszy kwestionariusz dotyczy Pana/Pani własnej oceny stanu biodra. Informacja ta pomoże nam zrozumieć, jakim problemem jest dla Pana/Pani biodro i w jakim stopniu może Pan/Pani wykonywać codzienne czynności. Proszę odpowiedzieć na każde pytanie, stawiając krzyżyk w wybranej i tylko jednej kratce dla każdego pytania. Jeśli nie jest Pan/Pani pewny/pewna jak odpowiedzieć, proszę zaznaczyć tę odpowiedź, która wydaje się najbliższa prawdy.

## Objawy

Poniższe pytania dotyczą objawów i trudności związanych ze stawem biodrowym, które występowały **w czasie ostatniego tygodnia.**

S1. Czy odczuwa Pan/Pani chrzęszczenie/tarcie lub słyszy przeskakiwanie lub jakikolwiek inny dźwięk w stawie biodrowym?

| Nigdy | Rzadko | Czasami | Często | Nieustannie |
|---|---|---|---|---|
| □ | □ | □ | □ | □ |

S2. Czy odczuwa Pan/Pani trudności w wykonaniu rozkroku?

| Żadnych | Niewielkie | Umiarkowane | Duże | Bardzo duże |
|---|---|---|---|---|
| □ | □ | □ | □ | □ |

S3. Czy odczuwa Pan/Pani trudności przy chodzeniu podczas stawiania kroków?

| Żadnych | Niewielkie | Umiarkowane | Duże | Bardzo duże |
|---|---|---|---|---|
| □ | □ | □ | □ | □ |

## Sztywność

Poniższe pytania dotyczą występowania sztywności (uczucie ograniczenia bądź spowolnienia możliwości wykonywania ruchu) stawu biodrowego **w ciągu ostatniego tygodnia.**

S4. W jakim stopniu nasilona jest sztywność w Pana/Pani stawie biodrowym rano, tuż po pierwszym wstaniu z łóżka?

| Brak sztywności | Niewielka | Umiarkowana | Duża | Bardzo duża |
|---|---|---|---|---|
| □ | □ | □ | □ | □ |

S5. W jakim stopniu nasilona jest sztywność Pana/Pani biodra po siedzeniu, leżeniu bądź odpoczynku w późniejszej porze dnia?

| Brak sztywności | Niewielka | Umiarkowana | Duża | Bardzo duża |
|---|---|---|---|---|
| □ | □ | □ | □ | □ |

## Ból

P1. Jak często odczuwa Pan/Pani ból stawu biodrowego?

| Nigdy | Raz w miesiącu | Raz w tygodniu | Codziennie | Cały czas |
|---|---|---|---|---|
| □ | □ | □ | □ | □ |

Jakie nasilenie bólu występowało u Pana/Pani **w ciągu ostatniego tygodnia** podczas wykonywania poniższych czynności stawu biodrowego?

P2. Pełne wyprostowanie biodra

| Brak bólu | Niewielkie | Umiarkowane | Duże | Bardzo duże |
|---|---|---|---|---|
| □ | □ | □ | □ | □ |

P3. Pełne zgięcie biodra

| Brak bólu | Niewielkie | Umiarkowane | Duże | Bardzo duże |
|---|---|---|---|---|
| □ | □ | □ | □ | □ |

P4. Chodzenie po płaskim podłożu

| Brak bólu | Niewielkie | Umiarkowane | Duże | Bardzo duże |
|---|---|---|---|---|
| □ | □ | □ | □ | □ |

P5. Wchodzenie i schodzenie ze schodów

| Brak bólu | Niewielkie | Umiarkowane | Duże | Bardzo duże |
|---|---|---|---|---|
| □ | □ | □ | □ | □ |

P6. W nocy podczas leżenia w łóżku

| Brak bólu | Niewielkie | Umiarkowane | Duże | Bardzo duże |
|---|---|---|---|---|
| □ | □ | □ | □ | □ |

P7. Siedzenie lub leżenie

| Brak bólu | Niewielkie | Umiarkowane | Duże | Bardzo duże |
|---|---|---|---|---|
| □ | □ | □ | □ | □ |

P8. Stanie w pozycji wyprostowanej

| Brak bólu | Niewielkie | Umiarkowane | Duże | Bardzo duże |
|---|---|---|---|---|
| □ | □ | □ | □ | □ |

P9. Chodzenie po twardym podłożu (asfalt, beton itp.)

| Brak bólu | Niewielkie | Umiarkowane | Duże | Bardzo duże |
|---|---|---|---|---|
| □ | □ | □ | □ | □ |

P10. Chodzenie po nierównym podłożu

| Brak bólu | Niewielkie | Umiarkowane | Duże | Bardzo duże |
|---|---|---|---|---|
| □ | □ | □ | □ | □ |

## Czynności życia codziennego

Poniższe pytania dotyczą sprawności fizycznej. Dzięki uzyskanym odpowiedziom będziemy mogli ocenić Pana/Pani zdolność do przemieszczania się i wykonywania codziennych czynności. Dla każdej z wymienionych czynności proszę określić stopień trudności, jaki wystąpił **w ciągu ostatniego tygodnia** przy jej wykonywaniu i który spowodowany jest stanem stawu biodrowego.

A1. Schodzenie ze schodów

| Bez trudności | Niewielki | Umiarkowany | Duży | Bardzo duży |
|---|---|---|---|---|
| □ | □ | □ | □ | □ |

A2. Wchodzenie po schodach

| Bez trudności | Niewielki | Umiarkowany | Duży | Bardzo duży |
|---|---|---|---|---|
| □ | □ | □ | □ | □ |

A3. Wstawanie z pozycji siedzącej

| Bez trudności | Niewielki | Umiarkowany | Duży | Bardzo duży |
|---|---|---|---|---|
| □ | □ | □ | □ | □ |

A4. Stanie

| Bez trudności | Niewielki | Umiarkowany | Duży | Bardzo duży |
|---|---|---|---|---|
| □ | □ | □ | □ | □ |

A5. Schylenie się/podniesienie przedmiotu z podłogi

| Bez trudności | Niewielki | Umiarkowany | Duży | Bardzo duży |
|---|---|---|---|---|
| □ | □ | □ | □ | □ |

A6. Chodzenie po płaskim podłożu

| Bez trudności | Niewielki | Umiarkowany | Duży | Bardzo duży |
|---|---|---|---|---|
| □ | □ | □ | □ | □ |

A7. Wsiadanie/wysiadanie z samochodu

| Bez trudności | Niewielki | Umiarkowany | Duży | Bardzo duży |
|---|---|---|---|---|
| □ | □ | □ | □ | □ |

A8. Robienie zakupów

| Bez trudności | Niewielki | Umiarkowany | Duży | Bardzo duży |
|---|---|---|---|---|
| □ | □ | □ | □ | □ |

A9. Zakładanie skarpet/pończoch

| Bez trudności | Niewielki | Umiarkowany | Duży | Bardzo duży |
|---|---|---|---|---|
| □ | □ | □ | □ | □ |

A10. Wstawanie z łóżka

| Bez trudności | Niewielki | Umiarkowany | Duży | Bardzo duży |
|---|---|---|---|---|
| □ | □ | □ | □ | □ |

A11. Zdejmowanie skarpet/pończoch

| Bez trudności | Niewielki | Umiarkowany | Duży | Bardzo duży |
|---|---|---|---|---|
| □ | □ | □ | □ | □ |

A12. Leżenie w łóżku (obracanie się, utrzymywanie pozycji biodra)

| Bez trudności | Niewielki | Umiarkowany | Duży | Bardzo duży |
|---|---|---|---|---|
| □ | □ | □ | □ | □ |

A13. Wchodzenie /wychodzenie z wanny

| Bez trudności | Niewielki | Umiarkowany | Duży | Bardzo duży |
|---|---|---|---|---|
| □ | □ | □ | □ | □ |

A14. Siedzenie

| Bez trudności | Niewielki | Umiarkowany | Duży | Bardzo duży |
|---|---|---|---|---|
| □ | □ | □ | □ | □ |

A15. Siadanie na sedes/Wstawanie z sedesu

| Bez trudności | Niewielki | Umiarkowany | Duży | Bardzo duży |
|---|---|---|---|---|
| □ | □ | □ | □ | □ |

A16. Wykonywanie cięższych prac domowych (przenoszenie ciężkich przedmiotów, czyszczenie podłóg itp.)

| Bez trudności | Niewielki | Umiarkowany | Duży | Bardzo duży |
|---|---|---|---|---|
| □ | □ | □ | □ | □ |

A17. Wykonywanie lekkich prac domowych (ścieranie kurzu, gotowanie itp.)

| Bez trudności | Niewielki | Umiarkowany | Duży | Bardzo duży |
|---|---|---|---|---|
| □ | □ | □ | □ | □ |

## Aktywność sportowa i rekreacyjna

Poniższe pytania dotyczą czynności związanych ze wzmożoną aktywnością fizyczną. W odpowiedziach należy określić stopień trudności wykonywania tych czynności ze względu na stan stawu biodrowego **w ciągu ostatniego tygodnia.**

SP1. Przysiady

| Bez trudności | Niewielki | Umiarkowany | Duży | Bardzo duży |
|---|---|---|---|---|
| □ | □ | □ | □ | □ |

SP2. Bieganie

| Bez trudności | Niewielki | Umiarkowany | Duży | Bardzo duży |
|---|---|---|---|---|
| □ | □ | □ | □ | □ |

SP3. Obracanie/skręcanie na obciążonej nodze

| Bez trudności | Niewielki | Umiarkowany | Duży | Bardzo duży |
|---|---|---|---|---|
| □ | □ | □ | □ | □ |

SP4. Chodzenie po nierównym podłożu

| Bez trudności | Niewielki | Umiarkowany | Duży | Bardzo duży |
|---|---|---|---|---|
| □ | □ | □ | □ | □ |

## Jakość życia

Q1. Jak często zwraca Pan/Pani uwagę na dolegliwości ze strony stawu biodrowego?

| Nigdy | Raz w miesiącu | Raz w tygodniu | Codziennie | Cały czas |
|---|---|---|---|---|
| □ | □ | □ | □ | □ |

Q2. Czy zmienił/zmieniła Pan/Pani swój styl życia mając na celu unikanie aktywności, które potencjalnie mogą prowadzić do uszkodzenia stawu biodrowego?

| W ogóle | Niewiele | Umiarkowanie | Znacznie | Całkowicie |
|---|---|---|---|---|
| □ | □ | □ | □ | □ |

Q3. Jak bardzo niepokoi Pana/Panią brak zaufania do własnego stawu biodrowego?

| W ogóle | Nieznacznie | Umiarkowanie | Poważnie | Bardzo poważnie |
|---|---|---|---|---|
| □ | □ | □ | □ | □ |

Q4. Jak duże trudności ogólnie sprawia Panu/Pani stan stawu biodrowego?

| Żadne | Niewielkie | Umiarkowanie | Poważne | Bardzo poważne |
|---|---|---|---|---|
| □ | □ | □ | □ | □ |

**Bardzo dziękujemy za dokładne wypełnienie ankiety.**
